# “Guide them to have a future orientation”: qualitative findings in developing a reinforcement-based intervention to address alcohol use with American Indian emerging adults

**DOI:** 10.3389/fpubh.2026.1745254

**Published:** 2026-04-08

**Authors:** Katherine A. Hirchak, Kellie Webb, Sharon Wagon, Kelsey Bajet, Allyson Kelley, James G. Murphy, Kamilla L. Venner

**Affiliations:** 1 Department of Community and Behavioral Health, Elson S. Floyd College of Medicine, Washington State University, Spokane, WA, United States; 2 Promoting Research Initiatives in Substance Use and Mental Health Collaborative, Washington State University, Spokane, WA, United States; 3 Connections in Indigenous Research, Cultural Leadership, Equity, & Solidarity, Washington State University, Spokane, WA, United States; 4 Tribal Healing Center, WY, United States; 5 Allyson Kelley & Associates, Sisters, OR, United States; 6 Department of Psychology, University of Memphis, Memphis, TN, United States; 7 Department of Psychology, Center on Alcohol, Substance Use & Addiction (CASAA), University of New Mexico, Albuquerque, NM, United States

**Keywords:** American Indian and Alaska Native communities, contingency management, emerging adults, qualitative, substance-free activities

## Abstract

**Background:**

The present study used qualitative research methods to assess interest in contingency management (CM; where incentives are provided after the return of an alcohol negative urine sample) enhanced with substance-free activities among emerging adults residing on a rural reservation. The research informs the future development and implementation of a clinical trial to address alcohol use among American Indian and Alaska Native (AI/AN) 18-29-year-olds.

**Methods:**

Semi-structured qualitative interviews and two focus groups were conducted at a rural reservation outpatient treatment healing center. Participant- relatives (i.e., study participants) were 18–29 years old and currently using alcohol or in recovery; family members of emerging adults who were currently using alcohol or in recovery; or Elders and healthcare professionals (*N* = 32). Applying the Healing Center Medicine Wheel (i.e., a framework that focuses on holistic health related to the physical, mental, emotional, and spiritual as well as the structural factors impacting health), a codebook was developed using a qualitative descriptive approach and data analyzed under the guidance of a Community Advisory Board (CAB).

**Results:**

Five themes were identified: (1) Importance of Culture and Ceremony; (2) Substance-Free Activities with Higher Levels of Enjoyment-Physical, Mental, Emotional and Spiritual; (3) Substance Free Activities: Barriers and Engagement; (4) Family Impact on Alcohol Use and Recovery; and (5) Views on Health and Recovery.

**Conclusion:**

Through community-driven strategies, frameworks and CAB guidance, a culturally responsive CM intervention was developed and is ongoing. This research provides a culturally informed process template for other Indigenous communities interested in addressing alcohol use among younger adults.

## Introduction

Rates of alcohol use among all youth have decreased over the past-ten years (1–3). Despite these trends, across racial and ethnic groups, more than 50% of AUDs are diagnosed before age 25 (4). Earlier alcohol use has been associated with long-term misuse and more severe AUD trajectory over the life course (5–7). While there are many cultural strengths across American Indian and Alaska Native (AI/AN) communities that are protective factors for alcohol misuse (8–10), relative to the general population, AI/AN youth initiate alcohol use at younger ages relative to non-AI/AN youth [e.g., (11–13)]. Younger AI/AN adults may also expereince disproportionately higher rates of binge drinking and alcohol use disorder (AUD) compared with their counterparts from other racial backgrounds (11). To effectively address substance use related inequities, research has underscored the importance of ensuring that interventions are culturally centered (14). Treatments that have reflected AI/AN beliefs, values and spiritualty have shown to improve engagement, retention (15), and program sustainability (16). There is a need for additional culturally appropriate, evidence-based interventions to address alcohol misuse among AI/AN emerging adults.

### Behavioral economics and contingency management

Behavioral economics (BE) views recovery from addiction as the change in patterns of behavior overtime, due to an increase in the accessibility and rewarding effects of substance-free activities (17). BE intervention approaches attempt to increase the salience of the longer-term negative impact of substance use on social, emotional, and physical functioning (e.g., the “response cost” associated with drinking). This is achieved through reducing delayed reward discounting and the relative reinforcing value of substance use (17). By reducing the relative reinforcing value of substance use, BE seeks to increase the importance of future goals and opportunities for engagement in substance-free activities to help people choose and sustain abstinence. Research has shown that participating in rewarding, substance-free activities is associated with decreased drinking among college students and women, and associated with greater motivations to reduce drinking across gender (18–20).

Contingency management (CM) has been conceptualized as an extension of BE (17, 21, 22). CM is one of the most effective interventions in the treatment of illicit substances and is effective across diverse populations (23–25). CM is a behavioral intervention based in operant conditioning that provides positive reinforcements for verified abstinence from drugs or alcohol (26–28). While decades of research evidence supports CM for substance use, barriers to real-world implementation have remained. These include regulatory challenges in providing incentives in healthcare, funding, workforce considerations and cultural acceptability (29). Previous research has suggested that CM may also address the same theoretical mechanisms of BE (e.g., realigning reinforcement contingencies) that have yielded reductions in alcohol use among college students and that these mechanisms within the context of CM should be explored further (30, 31).

To further increase the cultural acceptability of CM among AI/AN adults, previous research included conducting nine focus groups in several different AI/AN communities (32). Participants shared that CM was culturally congruent and suggested offering both practical (e.g., gift cards for gas) and cultural prizes (e.g., providing beading kits, a traditional artform consisting of patterned sewing of beads into fabric, leather or other materials) to enhance participation and support recovery. Results of focus groups completed with 18–29 year-olds and their families indicated that CM was appropriate to address alcohol misuse among younger generations (33). Specifically, offering prizes, cultural activities, and activities that capture the attention of young adults was identified as ideal for boosting CM engagement. Across communities, participants recommended marketing the intervention on social media and increasing community visibility and engagement through technology. Preliminary qualitative results of these CM studies are promising. However, the optimization of CM specifically for AI/AN 18–29 year olds has yet to be assessed. Given the success of interventions integrating the BE mechanisms of change among college students, there are opportunities to assess their applicability alongside CM among AI/AN populations.

### Present study

To culturally center a CM intervention integrating BE mechanisms of change among emerging AI/AN adults, a team of community and university researchers partnered on the present study. Through the convening of a Community Advisory Board (CAB) and completing qualitative interviews and focus groups, we examined 18–29 year olds’ experiences related to culture, participation in substance-free activities, spirituality and well-being in alcohol use recovery to better align the clinical trial with community need. This research is the first that we are aware of to design a CM enhanced intervention that is culturally centered for AI/AN 18–29 year olds. The research may have important public health implications for reducing alcohol related harms that disproportionately impact this population.

## Methods

### Ethical approval

The research took place on a reservation, that is home to two sovereign nations. Consistent with honoring Tribal sovereignty, two Tribal Business Councils reviewed and approved all research protocols, data collection instruments, and dissemination plans. The Washington State University Institutional Review Board [IRB; (#19027) and the Rocky Mountain Tribal IRB also approved all research protocols (PI: Hirchak, Webb)].

### Community-based participatory research, place, and positionality

Community-Based Participatory Research (CBPR) has demonstrated improved intervention engagement and health outcomes (34–36). Due to a strong community and research partnership spanning almost 15 years, a CBPR framework guided all aspects of the research process. A Community Advisory Board (CAB) was formed to provide guidance on research and processes. Six members joined to assist in re-centering the intervention form and content with traditional and cultural ways of thinking and evaluated the appropriateness of proposed program components. The CAB also guided the development of the focus group and interview questions and assisted with the interpretation of the results. This research was conducted at a healing center located on a reservation in the Northern Plains region of the United States. This reservation is characterized by its rural geography, rich landscapes, mountains, and rich cultural traditions and practices.

In alignment with reflexivity and the spirit of CBPR, the authors include a statement about their positionality (37). KH is a descendant of the Eastern Shoshone Tribe and has various European ancestry, mainly Italian. She has had research partnerships with AI/AN communities for 15 years. KW is Eastern Shoshone and Cowlitz with over 20 years of research and clinical experience. SW is an enrolled member of the Eastern Shoshone Tribe and has worked with her community for more than 20 years. KB is a descendant of immigrants and Filipino ancestry. She has worked with AI/AN communities for over 4 years. AK is a descendant of the Eastern Band of Cherokee Indians, as well as Swedish and Irish ancestry—she has worked with AI/AN communities for over two decades. JM is non-Indigenous and has meaningfully partnered with AI/AN communities on research, evaluation and public health initiatives for many years. KV is a member of the Native Village of Chitina Tribe and has about 30 years of experience partnering with AI/AN people on a range of research activities focused on substance use, treatment, and health and well-being.

### Community, participants and procedures

The Healing Center has operated on the reservation for several decades. They provide a range of cultural and spiritual activities consistent with the traditions of the Tribal communities in the region, in addition to evidence-based treatments for various substance use disorders. Activities within the community, and offered at the center, range from spiritual and ceremonial (e.g., Sundance, sweat lodge) to cultural (e.g., ribbon skirt making). Providers at the Healing Center shared recruitment materials at various community agencies, along with materials posted on community websites and social media. Prospective participants were screened for the following inclusion criteria: (1) self-identifying as an AI/AN 18–29-year-old who currently uses alcohol or is in recovery; (2) a family member of emerging adults (18+) who were currently using alcohol or in recovery; or (3) an Elder or healthcare professional (18+). Exclusion criteria included (1) IV drug use in the past year; (2) history of alcohol withdrawal (self-report); (3) traumatic brain injuries or other history of seizures (self-report); (4) illicit drug use primary over alcohol; (5) psychiatric conditions that preclude providing informed consent; and (6) previous participation in CM research (self-report). Inclusion criteria were based upon previous research with the community and CAB guidance (32, 33). No one was screened ineligible due to history of alcohol withdrawal, traumatic brain injury or psychiatric conditions.

The semi-structured interviews and focus groups included the same 12 questions. For example, we asked, “What are substance-free activities younger adults like to do?” For a complete list of interview questions, please see Supplementary Table S1. Interviews were conducted in-person or via video conference (i.e., Zoom) and lasted approximately 60 min. Participants were offered a $50 Tango gift card as compensation for time and effort, which was sent directly to their email.

### Data process, coding and analysis

Interviews and focus groups were recorded and then transcribed by a third party. About 30 % of the total transcripts were independently coded by two members of the research team. The recording of one interview accidentally ended approximately halfway through without the interviewer’s knowledge. Despite these missing data, we included this participant-relatives recorded and transcribed portion of the interview in our sample. We completed initial assessments of code application and frequency alongside an audit log for rationale and justification of the process; no additional intercoder reliability checks were completed. The codebook was entered into Dedoose software, and all data was coded. Frequency of applied codes across the sample was indicative of saturation. The coded data were then exported to Excel, and the lead author completed a thematic analysis (38).

Under the direction of the CAB, we used a hybrid process for developing the codebook. Both *a priori* and inductive coding were included in the final codebook. The Medicine Wheel has been used as a holistic framework for health and recovery from substance use in the existing literature for young adults (39, 40) the partnering community. It is a strengths-based approach that integrates the emotional, physical, mental and spiritual aspects of whole person wellbeing (41, 42). The CAB suggested that the Healing Center Medicine Wheel Framework be applied to the coding of the substance-free activity data, in alignment with both the literature and Indigenous worldviews (43). This included categorizing the substance-free activities into four domains, the physical, mental, spiritual and emotional. The Medicine Wheel is a central feature of the partnering Healing Centers’ whole-person treatment philosophy. Codes were discussed via email and in one-on-one meetings with CAB members and content experts in the field of treatment with AI/AN communities, until reaching a finalized codebook.

A word cloud was also developed from the data by counting the frequency and grouping of each activity mentioned by participant-relatives. Only activities mentioned more than once were included in the word cloud. The research team collapsed or combined activities by the Healing Center Medicine Wheel domains or if the activity essentially referred to the same thing (e.g., swimming, swimming in lakes, going to lakes or rivers). The results, including identified quotes and data interpretation, were presented to the CAB for further data analysis refinement.

## Results

### Participant characteristics

Two focus groups (*n* = 7) and 25 interviews were conducted. Participant-relatives’ ages ranged from 18 to 65 years old, with 34% of the sample aged 18–29. Many of the participant-relatives were female, and most were stably housed (Table 1).

**Table 1:** Sample characteristics.

Demographics	Participant-relatives
	*N* = 32
Age	38.3 (15.1)
Composition	
% of Sample that was 18–29 years old	34.0%
% Elders/providers	18.8%
Sex	
Female	68.8%
Male	31.3%
Education*	
Less than high school	18.8%
High school diploma or equivalent	37.5%
Some college/associate’s degree	38.5%
Bachelor’s degree	3.1%
Master’s/professional degree	3.1%
Employment	
Part-time	43.5%
Full-time	56.5%
Stably housed	98.0%

*Percentages may not total 100% due to rounding.

### Overview of themes

Five overarching themes were observed in these data. These include: (1) Importance of Culture and Ceremony, (2) Substance-Free Activities with Higher Levels of Enjoyment-Physical, Mental, Emotional, and Spiritual; (3) Substance-Free Activities: Barriers & Engagement; (4) Family Impact on Alcohol Use and Recovery; and (5) Views on Health and Recovery. Each theme includes a sub-theme presented with a corresponding quote.

### Theme 1. Importance of culture and ceremony

Culture and ceremony were highlighted as integral to health and well-being, as emphasized in the quotes in Table 2. Spirituality was discussed in the context of relationships with oneself, family and community. As described in *Connection*, participant-relatives spoke about connection to the land, and how the land holds ceremonial knowledge. Participant-relatives also consistently recognized the importance of Elders and the sacred (e.g., water; sub-theme *Ancestors & the Sacred*). Prayer was also mentioned frequently and was believed to increase well-being framed within the quadrants of the Healing Center Medicine Wheel. Many forms of spirituality were described. Broadly, participant-relatives mentioned spirituality in the context of cultural traditions, which were intended to keep the Tribe safe and enhance identity. For example, creation stories or other stories that carried meaning were meant to provide instruction for survival or group cohesion. While other ceremonial activities were highlighted as celebration (e.g., dances). Another aspect of culture and ceremony was related to how spirituality gives meaning, and that everyone is put on earth for a purpose. Substance use may disconnect one from their purpose, but that it can always be returned to as a source of strength.

**Table 2:** Culture and ceremony.

Subtheme	Exemplar quote
Connection	I was just always taught that you pray, you take care of yourself, that it’s okay to stumble and fall, and you give yourself credit. And that you just always want to reach out to your family, to whatever religion you have, reach out to who those people are, your Elders, and speak about it. And for myself and my family, we spent a lot of time outdoors and doing Sundances and ceremony…those kinds of things; they feed your spirit. (2009, 44 years old)
Ancestors & the sacred	…But within Native beliefs, the body is sacred, water is sacred, everything is sacred. And when you are putting alcohol in your body, you are poisoning yourself, essentially…it helps to know that your ancestors…ancestors play a huge role. And to know that your ancestors are looking down on you and that you want to make them proud, and you want to make your Elders proud. (2013, 22 years old)
Spirituality	You gotta be…well in every aspect, like, emotionally, mentally, physically and spiritually… Spirituality is above all three of those other ones because your spirit also needs to be fed. Our spirit needs prayers…whatever is ailing us mentally, physically, emotionally, take care of it with prayer. Where I see my well-being coming from is prayer, prayer centered. (2007, 51 years old)
Cultural celebration	Native Americans, they were big on feasts and just dinners, round dances we celebrate for people. Peyote meetings we celebrate for people. Maybe when they have been in recovery for a while, you could host a dinner, a feast, a meeting, a sweat, just to celebrate that they are coming back into the circle of life… A peyote meeting—or sweat. Those are really focused on celebration and helping… There’s sweats, powwows, all those things. Maybe creation stories. Those are really important to Tribes. (2014, 18 years old)

### Theme 2. Substance-free activities with higher levels of enjoyment: physical, mental, emotional and spiritual

**Figure 1:**
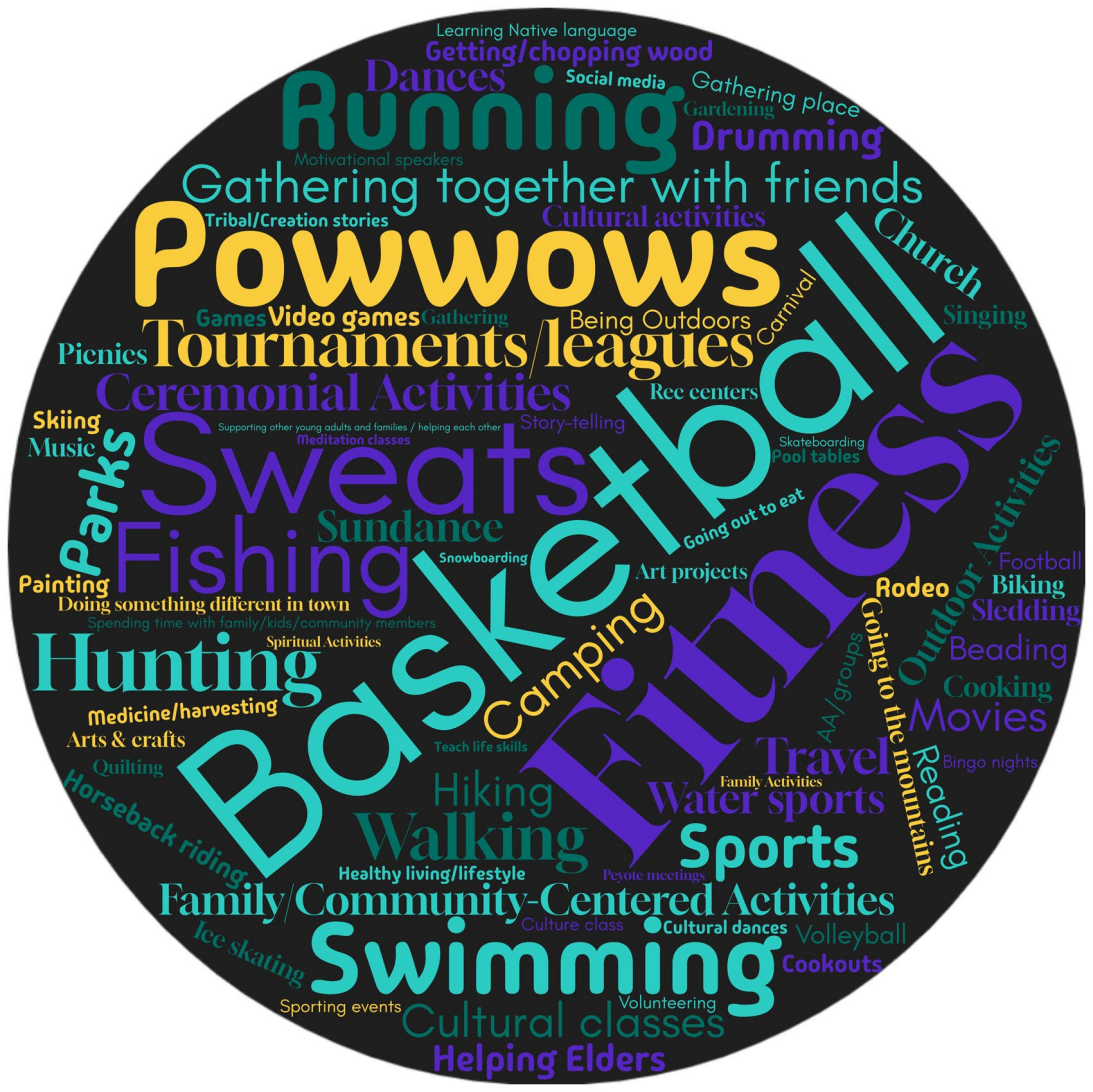
Top substance-free activities.

Enjoyable substance-free activities were discussed in each interview and focus group and also fell within the domains of the Healing Center Medicine Wheel (Figure 1). The highest rated activities were playing basketball, going to the gym, land-based or outdoor activities, and cultural engagement (e.g., beading). Level of enjoyment or activity type were also based upon the season (Table 3). Various activities were highlighted for winter (e.g., storytelling, learning from Elders, chopping wood for Elders), spring (e.g., volleyball), summer (e.g., powwows, swimming, going to the mountains), and fall (e.g., hunting).

**Table 3:** Substance-free activities with higher levels of enjoyment.

Subtheme	Exemplar quote
Seasonal sports	…in the wintertime, I have the volleyball league, and the basketball leagues…fall…hunting season. That’s the best time for everybody, and it keeps us well busy….that involves our horses…we always take up our horses. And then the springtime…it’s outside volleyball…four on four type of—volleyball. And then you get into some softball a little. Summer really leading into…roping…just rodeos all around. (1005, 24 years old)
Host cultural events	Like we had our regalia class here. That was great. We had families come and learn together… They helped each other make their dresses, their moccasins, and their belts. And that was fun to do that and watch them like, “Oh. I made that.” You know?… A round dance. Those are always good in the community. They have a good turnout when they have their round dances. (1001, Provider)

Some activities were believed to be compatible with alcohol use, such as drinking beer while fishing or at baseball games. While many of the more enjoyable or highly rated activities were deemed to be incompatible with alcohol use, including sweats or powwows, because of social norms prohibiting substance use and security at the event (Figure 1. Activity Word Cloud). Costs and infrastructure related to activities were also mentioned. A consistent suggestion for increasing access included building a recreational center where younger adults could meet and have a safe place to socialize. Overall, participant-relatives recommended expanding the availability of diverse low-cost activities offered in the community and providing transportation to events.

### Theme 3. Substance-free activities: barriers and engagement

Participant-relatives described a range of barriers and engagement strategies for recovery and treatment activities among younger adults (Table 4). Colonial determinants of health (i.e., structural racism and inequity that contribute to negative health and social outcomes) primarily consisted of a lack of transportation, employment opportunities, money, and available resources in the broader community that might assist younger people in achieving their goals. Trauma (e.g., sexual violence, death, post-traumatic stress disorder) was noted as a factor in substance use. Almost equally, mental health such as anxiety and depression was also mentioned as contributing factors for younger people choosing not to engage in healthy activities.

**Table 4:** Substance-free activities: barriers and engagement.

Subtheme	Exemplar quote
Importance of role models	…Mentor. Like being a sponsor to them for things about being in recovery. Taking ‘em to places like movie, dinners, to the horses and activities. And…physically, like running and riding a bike… When you are doing activities and things… Try talking to them in a positive way. And asking ‘em what do you think or asking—tell ‘em you care about us, stuff like that. They’ll trust you more, too. (Male FG)
Colonial determinants of health	Denial. Poverty. Not a lot of opportunities. Just sometimes it’s just pure hopelessness. They do not think that they have anything to look forward to. (1002, Elder)
Engagement strategies	Give them support. Support, transportation—I mean, everything that you have to do to make them successful. Offer it to them. Giving them rides to meetings, and—like, incentives like, ‘hey, I’m gonna help you’—because some people do not even have food in their homes… They-they cannot even pay their bills, so how are they gonna be like, ‘oh, let us stay on this road of recovery when my family—I cannot even take care of my family at home.’…Let us have case management, teach them how to be successful, how to live the right way… When you send these people home and they cannot even feed their family, it’s easier for them just to pick up a beer… (1008, 49 years old)
Family oriented & free	If they have kids already, make it a family thing. Like, ‘Oh, well. You can go and you can bring your kids with you and all.’ …‘Oh, wow. You should do this…and it’s free…’ That’s-that would be more engaging for other people because a lot of people, they do not got money… But stuff that-that is free for the community, a lot of people would be more engaged. (Female FG)
Goal setting & community recognition	Maybe just having like goals in life. More like fun goals. Setting big goals for themselves. Education, getting their degree or graduating, and then they could…have a better job. The family—well…the community, they—if, like, they graduate, then they get stuff from our Tribe… During our yearly powwow, when people—when they graduate from either college or high school, they have at the powwow where they honor them and then give ‘em Pendleton blankets and mention their name during the powwow…congratulate them. (2026, 24 years old)

At the other end of the continuum, strategies for engagement were framed at the individual, friend group, family, and community levels. Individual factors included younger adults’ interest and motivation to be in recovery. This was believed to be a personal choice and that recovery support needed to be tailored to each person and their needs. Engagement was also argued to be supported by external factors such as family and community. One example that was mentioned by most of the participant-relatives’ was the importance of future goal setting including going to college, vocational training and enhancement of career opportunities on the reservation or through the Tribe.

Time with family was highlighted as an enjoyable, substance-free activity. One individual referred to spending quality time with their parents and extended family as “medicine.” Participant-relatives also argued that many younger adults were likely to have children of their own, necessitating the need for broader family engagement strategies. This might take the form of various community activities open to all ages, like a free family portrait night. In addition, many participant-relatives believed that 18–29 year olds would benefit from role models or mentorship to help them achieve their goals, impart important life skills, and healthy coping strategies. It was recommended that this person either be someone that the young adult could relate to, healthy friends, or someone who is currently in recovery.

### Theme 4. Family impact on alcohol use and recovery

Across all the interviews, the impact of family was discussed. Most participant-relatives discussed how family is integral to young peoples’ health and recovery, framed as both supportive and potentially negative. As illustrated in the quotes in Table 5, some participant-relatives spoke of how encouraging and meaningful their families were in their own lives and how crucial this is for 18–29 year olds broadly. Participant-relatives underscored how families lend guidance and social support that can provide alternatives to substance use. Intergenerational support was also emphasized, with the care that grandparents and extended networks within the community can provide. A few participant-relatives highlighted potential ways that families may create unhealthy environments that could lead to generational challenges, including coping strategies of substance misuse and families drinking together. Regardless, participant-relatives agreed that having a supportive and strong family was the root of recovery and well-living.

**Table 5:** Family impact on alcohol use and recovery.

Subtheme	Exemplar quote
Positive family support	…But, if an individual grows up in a healthy home, with compassion and respect and all these other aspects of life that we consider positive, we could—you start to see the positives… I feel like that’s what creates, like, leaders; it’s just all these other aspects, like generosity, just carrying that wisdom, that honesty, understanding where they come from… And it’s also…a chain effect. When they develop their own families, they know how to create healthy structures… (1006, 23 years old)
Negative family influence	Their careers, being able to leave the reservation, get educated, possibly come back. But there are…strong family ties that they do not want to leave their huge families. And so that’s kind of their comfort zone. And sometimes families are enablers. They make it very easy to not go off to college, to not leave the reservation, to not need or want a job. (2016, 54 years old)

### Theme 5. Views on health and recovery

Health and recovery were described as both a benefit for the individual and the community. On the individual side, self-care examples included the revitalizing aspect of connecting with nature (Table 6). Recovery was also seen as personal in that it allowed individuals to return to themselves and take control of their lives. Participant-relatives also shared the many reasons why people might use substances such as family or community environment, friend group, mental health, trauma, and how alcohol impacts physical health. Given the many factors encompassed in recovery, the Healing Center Medicine Wheel was again referenced and that each domain needed to be in balance to optimize recovery. On the community side, recovery was seen as a path for individuals to become a part of their community again and give families their relatives back.

**Table 6:** Views on health and recovery.

Subtheme	Exemplar quote
Wellness	Being able to be connected with nature, I feel like, is also good because it’s always good to be in nature and good to be around, like, the beautiful trees and all that… We use medicine that helps us and heals us. That really does better people’s wellness. They usually, like, go from being not the strongest to being strong enough to make it to what they want to achieve. (2015, 19 years old)
You get your power back	…Whenever you are drinking and doing substance, you lose your power. You become defeated. You’re no longer yourself… But when you are sober, you defeated that, and you get your power back and you are no longer powerless. And it’s something that makes me feel great about. And, yeah, that’s what recovery and sobriety means to me — is being powerful. (2021, 31 years old)

With respect to health, examples included the cultural and spiritual (e.g., meditation), alongside the physical components such as personal hygiene, nutrition, and exercise. Fostering a good work ethic was mentioned, starting with how to run a household, complete chores and manage finances. Health and recovery were therefore defined within the context of holistic well-being, with reduction in alcohol use or alcohol abstinence merely one component and that a whole-person approach was necessary.

## Discussion

We completed 25 interviews and two focus groups on a rural reservation to determine the role of cultural engagement, spirituality, and how views on health shape alcohol use recovery. We also assessed enjoyable substance-free activities evaluated through the domains of the Healing Center Medicine Wheel including the Physical, Mental, Emotional and Spiritual. The qualitative research presented here was used to inform the design and implementation of a 12-week substance-free activity enhanced CM intervention for hazardous alcohol use among 18–29 year olds that is currently in progress. Qualitative results were integrated into the final design such as barriers and strategies to engagement (e.g., providing transportation), connecting participant-relatives to cultural substance free activities in the community, and inviting children and family to activities. This is the first collaboration that we are aware of to assess culturally centering various positive reinforcement strategies to encourage alcohol abstinence among younger AI/AN adults.

In line with the principles of behavioral economics, many of the responses focused on substance-free activities with an emphasis on events that were fun, and thus more likely to increase alcohol abstinence. Activities included powwows, sweats, land-based and family-oriented activities. Research with both AI/AN emerging adults (44, 45), and urban AI/AN adolescents (46), are consistent with our findings. Studies have suggested that engagement and access to substance-free activities that are cultural, including family or broader social connection, and are outdoors, are important to prevention and treatment of alcohol and other substance use (14, 44–48). To facilitate access, studies with AI/AN adolescents have hosted cultural activities for urban youth to attend (46). Another study for emerging AI/AN adults that was focused on a culturally grounded approach, integrated technology to increase engagement. The benefits of the use of technology included cutting down on gas and travel time (49). Collectively, the research points to the protective nature of the integration of traditional and evidence-based practices to promote substance use reductions for emerging adults.

Among non-AI/AN young adults, The Substance-Free Activity Session (SFAS) is another example of an approach that motivates engagement in recovery by focusing on interests and goals beyond substance use (18). In SFAS, principles of BE are integrated through personalized feedback and motivational interviewing to support young adults in goal setting with an eye towards the future (e.g., going to college). Emphasizing goals and engagement in rewarding substance free activities has led to increased alcohol abstinence (18). Therefore, it may also be viable to tangibly incentivize young AI/AN people with elevated alcohol use to engage in constructive activities as part of a CM approach.

Our preliminary qualitative findings also support the broader literature that AUD interventions incorporating BE mechanisms of change may further enhance recovery outcomes (18, 50). In a qualitative study completed with a non-Indigenous population assessing substance-free activities that were the most reinforcing, Meshesha and colleagues (51) highlighted self-care (e.g., prayer) and acts of service (e.g., volunteering) as among some of the most enjoyable. Participant-relatives in our study also noted these as important, framed as health and wellness, ceremony and spirituality in recovery. Additional research into strategies that enhance the reinforcing effects of activities during treatment are needed.

As with most studies, there were strengths and limitations to our approach. While consistent with the experiences of younger adults residing in the rural reservation community from which the partnership was based, our finding may not generalize to other Indigenous populations. Inclusion criteria were also based upon self-report, previous research in the community, and recommendations from the CAB, possibly limiting the broader clinical application of our work. A primary objective of the research was to determine potential strategies and cultural activities that enhance recovery from alcohol, from individuals in recovery and those currently drinking at levels they considered to be harmful. This could further limit the generalizability of what was reported here. In addition, our research was a collaboration with participant-relatives across culturally distinct communities. Differences exist in service delivery among Tribes. Some communities only engage in health initiatives specific to their Tribal members, which may further limit the application of our findings.

We also had challenges with recruitment due to COVID-19. Ultimately, we had less engagement in our focus groups both in number of participant-relatives, and in number of groups, than originally planned. However, other studies have highlighted the ability to obtain saturation with fewer participants in each group and wither fewer groups overall (52). We recruited additional participants to complete individual interviews to compensate for fewer focus groups.

The CAB also noted the high rates of employment and housing in the sample that may not reflect all individuals residing on the reservation. The demographics may have been due to the inclusion of service providers and participant-relatives’ with dual roles (e.g., both 18–29 and a service provider). We were also able to assess the internal validity of our data through conversations with the CAB about the codes, themes and their expertise in the interpretation of the results. The long-term collaboration between the community Healing Center, the CAB and the university researchers demonstrates the importance of integrating the principles of CBPR for partnership success.

## Conclusion

Through community-driven strategies, the use of a traditional framework for holistic health (i.e., the Medicine Wheel) and CAB guidance, these results of substance-free activities and barriers and facilitators of engagement in recovery provide the foundation for a culturally and developmentally responsive CM intervention for this community. This research also provides a process template informed by Indigenous knowledge that may be used by other AI/AN communities interested in addressing alcohol use among younger adults.

## Data Availability

The datasets presented in this article are not readily available because data ownership is maintained by the partnering Tribal communities. Requests to access the datasets should be directed to katherine.hirchak@wsu.edu.
