# Supplementary material for: “Guide them to have a future orientation”: qualitative findings in developing a reinforcement-based intervention to address alcohol use with American Indian emerging adults
Source: Front Public Health. 2026 Apr 8;14:1745254. doi: 10.3389/fpubh.2026.1745254 (PMC13099775; doi:10.3389/fpubh.2026.1745254)
Supplement: Supplementary file 1 [file Table_1.DOCX]

1. What are substance-free activities younger adults like to do? Or “I have the most fun when ____.”
2. Please rate how enjoyable each activity is.
3. Does the level of enjoyment change based upon the season (winter, spring, summer, fall)?
4. How could we support younger peoples’ engagement in substance-free activities? Or what might you suggest to help young people have healthy fun?
5. Are there barriers to engagement related to environment (e.g., SDOH: costs, housing, employment, transportation)?
6. What free/low-cost activities currently exist in the community for young people to do?
7. What barriers exist for young people to engage in healthy fun (e.g., SDOH: cost, housing, employment, transportation)?
8. What makes young people look forward to the future?
9. What are the strengths/good things about the young person’s family or community that support this?
10. How could this support be increased to help youth achieve their positive life goals?

Integrating Technology

As part of the intervention, we will have one group of young adults engage in 21 days of daily morning check-ins for their alcohol use and engagement in substance-free activities. Initially, we would like to include:

- Text reminders for check-ins
- Ability to make appointments at ESR online
- List of substance-free activities/interactive info around available activities
- Real time logging of data (uploading pictures of activities)

1. Do you like these features? Why/why not? What other features would you like to see?
2. Think apps that provide resources to meet needs, show what info is required to access resources, and other features within the app (e.g., A housing shelter app that provides shelter locations, availability, wait times, requirements for housing)
3. Do you have suggestions for the name of the intervention/app/website?
4. Images/symbols that reflect recovery

Spirituality and Culture

Now we are going to ask you questions about spirituality and culture.

1. How does spirituality/culture impact how Native people take care of themselves? (For example: holistically – mind, emotions, physical, spiritual?) Or “I feel most connected spiritually when I ____.”
2. How does substance use affect spirituality/culture? How does spirituality affect substance use? (Prompts: How does substance use impact/affect praying, dancing, participating in ceremony?)
3. Is there anything else you would like to share about changing counseling interventions to be more in line with Native ways and culture, more in line with your age group?

Models of Health

1. What were you taught about wellness or well-being? Do you still practice what you were taught/view it differently now?
2. How can we present cultural/traditional ideas of health to young people struggling with alcohol use? Or “I feel healthy when I _____.” Or “I feel hopeful about the future when___”
3. How can we support young adults’ future goals? Help them look forward to the future and being healthy?
4. What prevents younger people from being healthy (e.g., *personal*: depression, anxiety, trauma, etc.; *environmental*: lack of employment opportunity, etc.),

Recovery

1. What does recovery mean to you/your family/your Tribe/your community? Do you think this is important? Why, or why not?
2. What are the best ways to help people recover from misusing substances? For providers: What has helped clients you have worked with?

**If there’s time, ask:**

1. Contingency Management incorporates positive rewards as part of the structure of the program to encourage a person with addiction to be sober and get help for addiction. What are positive rewards that are consistent with Native culture? (Example, cooking favorite meal; visiting a sacred place)
2. What are examples of how we can reward our loved ones when they are sober?
3. How can we encourage loved ones to take better care of themselves (Example, perhaps in terms of the client’s relationship with their loved one who has an addiction)?
4. What are the usual ways that Native people go about asking other Eastern Shoshone/Native people to make changes? (For examples, rules or customs that may involve, gender, age, familial relationship, clan, religious society, tribal leader positions)
5. Are there Native stories or teachings that we could bring into Contingency Management/Behavioral Economics that are allowable/acceptable to share? (e.g., stories of family relationships; stories of helping each other; stories of setting limits; stories of how to talk with each other; stories of values)
6. What causes young people to use alcohol?
7. How do you think people start to misuse substances, is there a process for how that usually happens? (Prompt: Some people think addiction is caused by bad genes, trauma, or stress. What do you think causes addiction?)
